# Power imbalances in tropical medicine journals: an analysis of editorial board representation

**DOI:** 10.1186/s41182-025-00752-2

**Published:** 2025-07-11

**Authors:** Sushree Nibedita Panda, Manish Barik, P. Ratna, Prabhu Kalyan Das

**Affiliations:** 1 The George Institute for Global Health, Hyderabad, India; 2 Independent researcher, Delhi, India; 3 KIIT School of Public Health, KIIT University, Bhubaneswar, Odisha India; 4 ICMR Regional Medical Research Center, Bhubaneswar, Odisha India

**Keywords:** DEI, Tropical diseases, Global health and journals, Editorial boards

## Abstract

**Background:**

Shaped by its colonial origins, tropical medicine sustains inequitable power dynamics in global health, sidelining low-middle-income countries (LMICs) in critical decision-making processes over research agendas and priorities. Editorial boards of tropical medicine journals, dominated by scholars from high-income countries (HICs), risk reinforcing power imbalances and excluding context-driven expertise from endemic regions. This study examines the diversity of editorial boards across gender, geographic, socioeconomic, and geopolitical dimensions to assess systemic inequities.

**Method:**

A systematic search of the National Library of Medicine (NLM) catalog was conducted via a targeted strategy between October and December 2024. After screening 153 journals for title relevance and applying exclusion criteria based on publication status, availability of editorial information, and global scope, 24 journals were selected. Data on 2,226 editorial board members were extracted from journal and institutional websites. Data on gender, country of affiliation (classified by World Bank income/regions), and geopolitical groups (G7, G20, BRICS) were extracted from public sources. Gender determination used a sequential approach (journal descriptions, Genderize.io, and consensus). Descriptive statistics were used to perform the analysis.

**Results:**

The editorial board comprised 2,226 members, 66% male, 31.2% female, and 2.8% undetermined, from 120 nations. The regional contributions included Europe and Central Asia (21.9%), North America (20.9%), East Asia and the Pacific (16.6%), and Latin America and the Caribbean (16.2%), whereas Sub-Saharan Africa (11.2%), South Asia (9.7%), and the Middle East and North Africa (3.4%) were underrepresented. Over half (52.8%) were affiliated with high-income countries. Geopolitically, 40.3% were from the G7, 67.1% were from the G20, and 24.2% were from the BRICS. Some journals showed skewing, with 85.2% North American representation and 90.3% East Asia–Pacific dominance.

**Conclusion:**

Tropical medicine editorial boards are steeped in systemic inequities that echo colonial legacies, with the overrepresentation of HICs and men limiting LMIC perspectives and local expertise. This imbalance undermines research relevance and ethical integrity by prioritizing Global North agendas over the needs of populations most affected by tropical diseases. To address these disparities, substantial reforms are essential. Strategies such as instituting DEI (Diversity, Equity and Inclusion), creating targeted mentorship programs for LMIC researchers, and enforcing transparent, bias-resistant recruitment practices are important. Such measures will create a more inclusive editorial landscape that aligns research priorities with global health needs, promoting equitable and contextually relevant solutions.

**Supplementary Information:**

The online version contains supplementary material available at 10.1186/s41182-025-00752-2.

## Introduction

The term “tropical medicine” originated in the nineteenth century as a colonial construct aimed at protecting European colonizers from diseases prevalent in colonized regions [1]. Embedded in notions of otherness and racial superiority, the term reinforces a dichotomy between the “tropical” (framed as exotic, dangerous, and distant) and the Global North, continuing epistemic hierarchies that marginalize low- and middle-income countries (LMICs) [2]. This framing risks trivializing tropical diseases as geographically confined issues, despite evidence that climate change, migration, and global inequities have expanded their reach beyond traditional tropical zones [3, 4].

Global funders allocate 75% of direct and 70% of indirect neglected tropical disease (NTD) funding to leading institutes in nonendemic nations, modernizing infrastructure while perpetuating colonial legacies through sample collection in low-income countries [5]. Such narratives, rooted in interpretive and credibility biases, justify imposing nonendemic institutional oversight, which complicates governance without increasing accountability. Such practices create power imbalances, relegating endemic nations to passive subjects rather than partners in research addressing diseases that disproportionately affect their populations [6, 7]. This also marginalizes endemic country researchers, denying them leadership roles and creating perceptions of a lack of “merit” to shape global tropical disease agendas. Concurrently, local experiential knowledge—such as ethnobiological insights from indigenous communities—is dismissed as nonscientific, despite its nuanced understanding of disease ecology [8–10]. This epistemic exclusion exemplifies testimonial injustice: privileging narrow “objective” frameworks over context-rich, community-driven evidence essential for equitable solutions.

Journals are widely regarded as “duty bearers” of core ethical standards, playing a pivotal role in ensuring that both newly produced and disseminated knowledge maintains its ethical integrity [11]. Editorial boards of leading journals—critical gatekeepers of knowledge production—remain dominated by scholars from high-income, nonendemic countries. This exclusion of experts from disease-endemic regions entrenches epistemic injustice, sidelining those with lived experience of the poverty‒disease nexus and narrowing the scope of “legitimate” scientific expertise [12]. Such homogeneity risks prioritizing research agendas aligned with Global North interests while overlooking context-specific challenges in LMICs [13, 14].

Diverse editorial boards are not merely symbolic; they directly shape research inclusivity and relevance. Representations from LMICs, endemic regions, and marginalized genders broaden the perspective of peer review, improve fairness in manuscript selection, and amplify submissions from underfunded regions. However, while gender disparities in medical editorial boards have been documented across specialties, tropical medicine journals remain understudied [15–18]. Addressing this gap is urgent: inclusive governance is vital in dismantling colonial legacies and ensuring that research reflects the needs of those most affected by tropical diseases. This study aims to understand the extent of diversity in the editorial boards of Tropical Medicine journals.

## Methods

In this cross-sectional study, we searched the National Library of Medicine (NLM) Catalog (https://www.ncbi.nlm.nih.gov/nlmcatalog/) to find relevant journals in Tropical Medicine via the search strategy given in Supplemental Table 1

During the first phase, the journals were screened based on the relevance of their titles. Journals not relevant to tropical medicines were subsequently excluded. The remaining journals were further screened in the second phase via an elaborate eligibility criterion. Journals were excluded for the following reasons: (1) publication ceased; (2) no information from editorial boards; (3) no global scope; (4) not a medical or scientific journal; and (5) no records of the journal were found. To assess eligibility, we reviewed the aims and/or scopes, as stated on their respective journal websites.

**Fig. 1:**
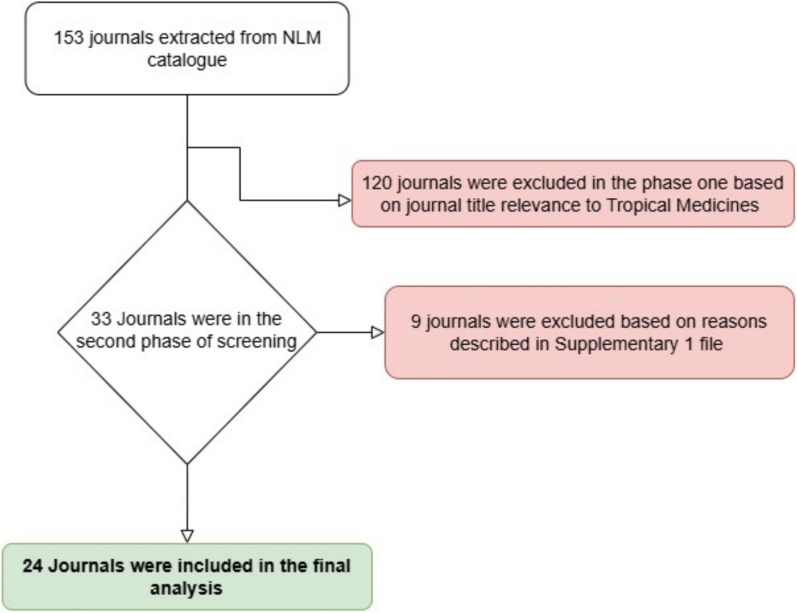
Selection of journals

We found 153 journals that publish tropical disease-related research. We excluded 120 journals from the journal title and 9 journals by reviewing the aims and scopes of the journals. Finally, 24 journals were included in the analysis. A detailed flowchart of the selection process is given in Fig. 1. A list of excluded journals with reasons is provided in Supplemental Table 2.

The data were collected by three authors (PKD, PR and SNP) and were later cross-checked for consistency by MB. We extracted information on the members of the editorial board from official resources of journals or their institutional profiles available in the public domain between October 2024 and December 2024.

We focused our analysis on the following aspects of representativeness:

- Country income status
- Geopolitical grouping
- geographic regions and
- gender (men and women are impacted differently).

We determined the gender and country of primary work affiliation via an approach that has been previously published and used to assess diversity in editorial boards of global health journals [18] and was later used extensively for assessing diversity across several domains by others [19–21]. Gender was assessed in a binary (“male” and “female”) manner (while we encourage analyses to include nonbinary people, the data and methods restricted us from doing so here), using a sequential approach (i.e., by going to the next criterion if the preceding one did not yield enough information): first, the editors’ description on the journal website; then, second, a web application, Genderize (https://genderize.io/), which determines the gender of a first name along with a probabilistic certainty score (accepted only when ≥ 0.95); then, a description on the website (pictures/ pronouns or any other clues of gender; and finally, an inspection of names by the authors (decided by consensus).

We used a sequential approach to identify the country in which editorial members listed their primary employer or affiliated organization: first, descriptions on the journal website; then, member descriptions on the institutional website. For gender and country, if none of the steps led to an inference, we marked the category as undetermined (unknown) for the editorial board members. We classified the country affiliation using the World Bank classification of income status into four groups: “low-income countries (LICs)”, “lower-middle-income countries (LMICs)”, “upper-middle-income countries (UMICs)” and “high-income countries (HICs) [22]. We also divided the countries according to the World Bank region classifications into 7 groups: North America (NA), East Asia and Pacific (EAP), Middle East and North Africa (MENA), South Asia (SA), Europe and Central Asia (ECA), Latin America and the Caribbean (LAC), and sub-Saharan Africa (SSA). The country affiliation of each included editorial board member was categorized into the following political‒economic categories: “G20”. “G7”, “BRICS” and “none”.

The collected data were organized via Microsoft Excel (Microsoft 365), and after being categorized into distinct variables, a descriptive analysis was performed to illustrate the composition and background of members across 24 journals. The analysis relied upon reviewing publicly available information on journal websites and/or institutional webpages.

### Ethical consideration

Since only data available in the public domain (from the journal and institutional websites) were used, and the study did not involve any living participants, due to which ethical clearance was not needed.

## Results

A total of 24 Tropical Medicine journals with 2226 editorial board members were included in this analysis. Table 1 presents a comprehensive list of journals included in this study, detailing the name of each journal, a direct link to its editorial board webpage, and the total number of board members. Smaller boards are seen with *Leprosy Review* (12 members) and *Research and Reports in Tropical Medicine* (10 members). Remarkably, *Frontiers in Tropical Diseases* stands out with 881 board members, and *PLOS Neglected Tropical Diseases* follows with 425 board members, emphasizing a broad scale in editorial governance. There were a total of 2226 editorial board members from 120 nations included in the analysis, and the USA had the highest number of editorial board members (19.5%), followed by Brazil (9.3%), India (7.9%), and the UK (6.6%).

**Table 1:** List of journals included in this study

Serial no	Name of the Journal	Link to the journal	No of board members
1	Acta Tropica	https://www.sciencedirect.com/journal/acta-tropica/about/editorial-board	61
2	The American Journal of Tropical Medicine and Hygiene	https://www.ajtmh.org/page/editorialboard	27
3	Annals of Tropical Medicine and Public Health	https://www.scienceopen.com/collection/8bc5167b-b1c0-40e2-bd42-a12e59e8ef49	20
4	Asian Pacific Journal of Tropical Biomedicine	https://journals.lww.com/aptb/pages/editorialboard.aspx	92
5	Asian Pacific Journal of Tropical Medicine	https://journals.lww.com/APTM/pages/editorialboard.aspx	76
6	Current Tropical Medicine Reports	https://link.springer.com/journal/40475/editorial-board	18
7	Indian Journal of Dermatology, Venereology and Leprology	https://ijdvl.com/editorial-board/	50
8	Indian Journal of Leprosy	https://www.ijl.org.in/editorial-team	26
9	Journal of Tropical Medicine	https://onlinelibrary.wiley.com/page/journal/2720/homepage/editorial-board	23
10	The Journal of Venomous Animals and Toxins Including Tropical Diseases	https://jvat.org.br/about/index/editorial-board#335	23
11	JSM Tropical Medicine and Research	https://www.jscimedcentral.com/editorial-board/JSM-Tropical-Medicine-and-Research	38
12	Leprosy Review	https://leprosyreview.org/eboard	12
13	Parasites, Hosts and Diseases	https://parahostdis.org/about/editorial.php	44
14	Research and Reports in Tropical Medicine	https://www.tandfonline.com/journals/drrt20/about-this-journal#editorial-board	10
15	Transactions of the Royal Society of Tropical Medicine and Hygiene	https://academic.oup.com/trstmh/pages/Editorial_Board	52
16	Tropical Biomedicine	https://msptm.org/journal/	31
17	Tropical Diseases, Travel Medicine and Vaccines	https://tdtmvjournal.biomedcentral.com/about/editorial-board	45
18	Tropical Doctor	https://journals.sagepub.com/editorial-board/TDO	10
19	Tropical Medicine and Health	https://tropmedhealth.biomedcentral.com/about/editorial-board	31
20	Tropical Medicine and International Health	https://onlinelibrary.wiley.com/page/journal/13653156/homepage/editorialboard.html	55
21	Tropical Medicine and Surgery	https://www.walshmedicalmedia.com/tropical-medicine-and-surgery/editorial-board.html	42
22	Frontiers in Tropical Diseases	https://www.frontiersin.org/journals/tropical-diseases/editors	881
23	Tropical Medicine and Infectious Disease	https://www.mdpi.com/journal/tropicalmed/editors	135
24	PLOS Neglected Tropical Diseases	https://journals.plos.org/plosntds/static/editorial-board	425

The findings are also available at interactive dashboard format.

### Overall diversity of editorial board members

Table 2 shows disparities across genders, geographic origins, economic statuses, and political‒economic affiliations of editorial board members. Out of a total of 2,226 members, 1,469 (66%) were male, 694 (31.2%) were female, and 63 (2.8%) were undetermined, highlighting a pronounced gender imbalance. In terms of geographic distribution based on World Bank regions, members were notably diverse: the ECA contributed 488 (21.9%), North America contributed 466 (20.9%), the EAP contributed 370 (16.6%), and the LAC contributed 361 (16.2%). SSA and South Asia accounted for 250 (11.2%) and 215 (9.7%) members, respectively, whereas MENA represented a smaller share at 3.4%. In terms of economic classification, over half of the members (1,149; 52.8%) were HICs, 539 (24.8%) were UMICs, 425 (19.5%) were LMICs, and only 64 (2.9%) were LICs, reflecting a strong skew toward wealthier nations. Politically, the affiliations further underscore these trends: 897 members (40.3%) belong to the G7 nations, 1,493 (67.1%) are from G20 countries, and 514 (24.2%) are from BRICS countries.

**Table 2:** Diversity in Tropical Medicine Journals

	N, %
Gender	
Male	1469, 66
Female	694, 31.2
Undetermined	63, 2.8
World Bank regions	
Europe and Central Asia	488, 21.9
North America	466, 20.9
East Asia and Pacific	370,16.6
Latin America and the Caribbean	361,16.2
Sub-Saharan Africa	250, 11.2
South Asia	215, 9.7
Middle East and North Africa	76, 3.4
Country Income Level (World Bank classifications)	
High-Income Countries	1149, 52.8
Upper-middle Income Countries	539, 24.8
Low-middle Income Countries	425, 19.5
Low-Income Countries	64, 2.9
Political-economic affiliations of countries	
G7	897, 40.3
G20	1493, 67.1
BRICS	514, 24.2

**G7** countries (Canada, France, Germany, Italy, Japan, United Kingdom and United States of America)

**G20** (Argentina, Australia, Brazil, Canada, China, France, Germany, India, Indonesia, Italy, Japan, Republic of Korea, Mexico, Russia, Saudi Arabia, South Africa, Türkiye, United Kingdom and United States)

**BRICS** ( Brazil, Russia, India, China, South Africa, Egypt, Ethiopia, Indonesia, Iran and the United Arab Emirates.)

### Diversity by gender

Table 3 reveals a consistent predominance of male participants across most titles. *Acta Tropica*, *The American Journal of Tropical Medicine and Hygiene*, and *Annals of Tropical Medicine and Public Health* have higher male proportions of 70.5%, 74.1%, and 75%, respectively, while female representation ranges between 25% and 29.5%. Similarly, the *Asian Pacific Journal of Tropical Medicine* and the *Asian Pacific Journal of Tropical Biomedicine* show high male representation, nearing 79% and 85%, respectively, with corresponding lower female percentages. In contrast, the *Indian Journal of Leprosy* reported an exceptionally high percentage of males (92.3%), whereas *Leprosy Review* reported a higher percentage of females (58.3%). Journals such as *Current Tropical Medicine Reports* and the *Indian Journal of Dermatology, Venereology and Leprology* demonstrate a more balanced gender distribution. Furthermore, *Parasites, Hosts and Diseases* and *Tropical Medicine and International Health* include a notable “undetermined” category, at 50% and 9.1%, respectively.

**Table 3:** Gender representations in the Tropical Medicine journals

Name of the journal	Gender		
	Male (%)	Female (%)	Undetermined (%)
Acta Tropica	70.5	29.5	0
The American Journal of Tropical Medicine and Hygiene	74.1	25.9	0
Annals of Tropical Medicine and Public Health	75	25	0
Asian Pacific Journal of Tropical Medicine	78.9	21.1	0
Asian Pacific Journal of Tropical Biomedicine	84.8	15.2	0
Current Tropical Medicine Reports	55.6	44.4	0
Indian Journal of Dermatology, Venereology and Leprology	54	46	0
Indian Journal of Leprosy	92.3	7.7	0
Journal of Tropical Medicine	69.6	30.4	0
The Journal of Venomous Animals and Toxins Including Tropical Diseases	60.9	39.1	0
JSM Tropical Medicine and Research	71.1	28.9	0
Leprosy Review	41.7	58.3	0
Parasites, Hosts and Diseases	34.1	15.9	50
Research and Reports in Tropical Medicine	77.8	22.2	0
Transactions of the Royal Society of Tropical Medicine and Hygiene	71.2	28.8	0
Tropical Biomedicine	48.4	45.2	6.5
Tropical Diseases, Travel Medicine and Vaccines	60	37.8	2.2
Tropical Doctor	70	30	0
Tropical Medicine and Health	64.5	35.5	0
Tropical Medicine and International Health	69.1	21.8	9.1
Tropical Medicine and Surgery	78.6	21.4	0
Frontiers in Tropical Diseases	62.1	36.1	1.8
Tropical Medicine and Infectious Disease	79.3	20.7	0
PLOS Neglected Tropical Diseases	65.2	30.8	0

### Diversity by geographical region

Table 4 shows that several journals are heavily dominated by a single region. For example, 85.2% of the board members of the *American Journal of Tropical Medicine and Hygiene* are from North America, whereas *Tropical Biomedicine* (90.3%) and *Tropical Medicine and Health* (80.6%) are primarily represented by members of the EAP. Similarly, the *Indian Journal of Leprosy* is dominated by South Asia (84.6%), and *the Journal of Venomous Animals and Toxins Including Tropical Diseases* is largely composed of members from LAC (60.9%). In contrast, some journals exhibit a more balanced distribution. *Acta Tropica* draws members from the ECA (31.1%), EAP (19.7%), LAC (18%), and North America (13.1%), whereas the *Journal of Tropical Medicine* shows significant representation from the EAP (26.1%) and South Asia (21.7%), alongside other regions. A few journals, such as *Tropical Doctor*, highlight underrepresented regions, with 40% of its board from Sub-Saharan Africa yet lacking representation from North America, EAP, LAC, or MENA.

**Table 4:** Representation of Editorial Boards in Tropical Medicine Journals by World Bank region

Name of the journal	World Bank Classifications of Regions						
	EAP (%)	ECA (%)	LAC (%)	MENA (%)	SA (%)	NA (%)	SSA (%)
Acta Tropica	19.7	31.1	18	6.6	3.3	13.1	8.2
The American Journal of Tropical Medicine and Hygiene	7.4	0	7.4	0	0	85.2	0
Annals of Tropical Medicine and Public Health	0	5	0	30	30	5	30
Asian Pacific Journal of Tropical Medicine	27.6	34.2	5.3	5.3	11.8	9.2	6.6
Asian Pacific Journal of Tropical Biomedicine	20.7	29.3	7.6	9.8	5.4	10.9	16.3
Current Tropical Medicine Reports	0	0	16.7	11.1	5.6	55.6	11.1
Indian Journal of Dermatology, Venereology and Leprology	0	0	0	100	0	0	0
Indian Journal of Leprosy	0	7.7	0	0	84.6	7.7	0
Journal of Tropical Medicine	26.1	8.7	13	13	21.7	4.3	13
The Journal of Venomous Animals and Toxins Including Tropical Diseases	4.3	21.7	60.9	8.7	0	0	4.3
JSM Tropical Medicine and Research	10.5	15.8	26.3	13.2	0	23.7	10.5
Leprosy Review	16.7	50	0	0	0	33.3	0
Parasites, Hosts and Diseases	86.4	2.3	4.5	0	0	6.8	0
Research and Reports in Tropical Medicine	11.1	33.3	11.1	11.1	33.3	0	0
Transactions of the Royal Society of Tropical Medicine and Hygiene	23.1	32.7	3.8	1.9	13.5	25	1.9
Tropical Biomedicine	90.3	3.2	0	0	3.2	3.2	0
Tropical Diseases, Travel Medicine and Vaccines	15.6	24.4	8.9	4.4	4.4	37.8	4.4
Tropical Doctor	0	30	0	0	30	0	40
Tropical Medicine and Health	80.6	12.9	0	0	0	6.5	0
Tropical Medicine and International Health	14.5	60	3.6	0	5.5	5.5	10.9
Tropical Medicine and Surgery	26.2	14.3	4.8	7.1	0	47.6	0
Frontiers in Tropical Diseases	9.2	19	25.2	1.6	8.3	17.8	19
Tropical Medicine and Infectious Disease	26.7	38.5	5.2	1.5	0.7	19.3	8.1
PLOS Neglected Tropical Diseases	13.2	22.6	15.3	4.5	5.2	35.1	4.2

“North America(NA)”, “East Asia and Pacific(EAP)”, “Middle East and North Africa (MENA)”, “South Asia(SA)”, “Europe and Central Asia(ECA)”, “Latin America and the Caribbean (LAC)”, and “Sub-Saharan Africa(SSA)”

### Diversity country income status

Table 5 shows significant differences in the representation of editorial board members on the basis of World Bank income status. *Acta Tropica* comprises nearly half of its HICs (49.2%), with substantial representation from UMICs (36.1%) and LMICs (13.1%), and a minor contribution from LICs (1.6%). In contrast, *the American Journal of Tropical Medicine and Hygiene* is overwhelmingly composed of members from HICs (92.6%), with a small share from UMICs (7.4%). *Annals of Tropical Medicine and Public Health* present a notable deviation, with 65% of its board drawn from LMICs, complemented by members from high-income (25%) and upper-middle-income (10%) regions. Both the *Asian Pacific Journal of Tropical Medicine* and the *Asian Pacific Journal of Tropical Biomedicine* display balanced distributions, with approximately half of their representation coming from high-income regions. Journals such as the *Indian Journal of Dermatology, Venereology and Leprology* and the *Indian Journal of Leprosy* are predominantly focused on lower-middle-income areas (100% and 84.6%, respectively). Other titles, such as the *Journal of Tropical Medicine*, *the Journal of Venomous Animals and Toxins Including Tropical Diseases*, and *Tropical Doctor*, show mixed profiles, while many journals maintain a strong affiliation with HICs.

**Table 5:** Representation of Editorial Boards in Tropical Medicine Journals by World Bank Income Groups

Name of the journal	World Bank Income status			
	HICs (%)	UMICs (%)	LMICs (%)	LICs (%)
Acta Tropica	49.2	36.1	13.1	1.6
The American Journal of Tropical Medicine and Hygiene	92.6	7.4	0	0
Annals of Tropical Medicine and Public Health	25	10	65	0
Asian Pacific Journal of Tropical Medicine	48.7	30.3	18.4	2.6
Asian Pacific Journal of Tropical Biomedicine	50	23.9	19.6	6.5
Current Tropical Medicine Reports	66.7	11.1	11.1	11.1
Indian Journal of Dermatology, Venereology and Leprology	0	0	100	0
Indian Journal of Leprosy	15.4	0	84.6	0
Journal of Tropical Medicine	17.4	47.8	34.8	0
The Journal of Venomous Animals and Toxins Including Tropical Diseases	26.1	65.2	8.7	0
JSM Tropical Medicine and Research	42.1	42.1	13.2	2.6
Leprosy Review	91.7	0	8.3	0
Parasites, Hosts and Diseases	86.4	13.6	0	0
Research and Reports in Tropical Medicine	22.2	33.3	44.4	0
Transactions of the Royal Society of Tropical Medicine and Hygiene	67.3	17.3	13.5	1.9
Tropical Biomedicine	9.7	83.9	6.5	0
Tropical Diseases, Travel Medicine and Vaccines	73.3	13.3	13.3	0
Tropical Doctor	30	0	50	20
Tropical Medicine and Health	96.8	3.2	0	0
Tropical Medicine and International Health	69.1	12.7	16.4	1.8
Tropical Medicine and Surgery	66.7	31	2.4	0
Frontiers in Tropical Diseases	43	28.6	23.8	4.5
Tropical Medicine and Infectious Disease	77.8	17	3	2.2
PLOS Neglected Tropical Diseases	68.3	20.7	9.5	1.4

“low-income countries (LICs)”, “lower-middle-income countries (LMICs)”, “upper-middle-income countries (UMIC)” and “high-income countries (HICs)

### Diversity by geopolitical affiliation

In Table 6, *Acta Tropica* shows a balanced representation of 50% in the G7, 67.2% in the G20, and 60% in the BRICS. In contrast, *the percentage of the American Journal of Tropical Medicine and Hygiene* was only 50% in the G7 but was 96.3% in the G20 and 83.3% in the BRICS. Moreover, *Annals of Tropical Medicine and Public Health* favor the G7 (66.7%) over the G20 (45%) and BRICS (33.3%). The *Asian Pacific Journal of Tropical Medicine* and *Asian Pacific Journal of Tropical Biomedicine* presented moderate to high percentages across groups. Notably, the *Indian Journal of Dermatology, Venereology and Leprology* is exclusively aligned with the G20 and BRICS (100% each), whereas the *Indian Journal of Leprosy* has lower G7 (33.3%) and BRICS (33.3%) affiliations despite high G20 representation (92.3%). Other journals, including the *Journal of Tropical Medicine*, *Current Tropical Medicine Reports*, and *Tropical Medicine and Health*, revealed similar disparities.

**Table 6:** Representation of Editorial Boards in Tropical Medicine Journals by Geopolitical affiliations

Name of the journal	Geopolitical affiliations		
	G7 (%)	G20 (%)	BRICS (%)
Acta Tropica	50	67.2	60
The American Journal of Tropical Medicine and Hygiene	50	96.3	83.3
Annals of Tropical Medicine and Public Health	66.7	45	33.3
Asian Pacific Journal of Tropical Medicine	50	56.6	62.5
Asian Pacific Journal of Tropical Biomedicine	50	45.7	36.4
Current Tropical Medicine Reports	50	77.8	81.8
Indian Journal of Dermatology, Venereology and Leprology	0	100	100
Indian Journal of Leprosy	33.3	92.3	33.3
Journal of Tropical Medicine	33.3	64.2	50
The Journal of Venomous Animals and Toxins Including Tropical Diseases	50	65.2	71.4
JSM Tropical Medicine and Research	50	71.1	50
Leprosy Review	50	68.7	0
Parasites, Hosts and Diseases	50	88.6	20
Research and Reports in Tropical Medicine	33.3	77.8	33.3
Transactions of the Royal Society of Tropical Medicine and Hygiene	50	78.8	16.7
Tropical Biomedicine	25	19.4	25
Tropical Diseases, Travel Medicine and Vaccines	40	55.6	25
Tropical Doctor	50	40	20
Tropical Medicine and Health	66.7	96.8	0
Tropical Medicine and International Health	50	50.9	9.1
Tropical Medicine and Surgery	50	76.2	14.3
Frontiers in Tropical Diseases	50	62.3	24.9
Tropical Medicine and Infectious Disease	50	71.9	12.6
PLOS Neglected Tropical Diseases	50	24.2	21.2

**G7** countries(Canada, France, Germany, Italy, Japan, United Kingdom and United States of America)

**G20** (Argentina, Australia, Brazil, Canada, China, France, Germany, India, Indonesia, Italy, Japan, Republic of Korea, Mexico, Russia, Saudi Arabia, South Africa, Türkiye, United Kingdom and United States)

**BRICS** (Brazil, Russia, India, China, South Africa, Egypt, Ethiopia, Indonesia, Iran and the United Arab Emirates.)

## Discussion

The analysis of 24 tropical medicine journals reveals systemic inequities in editorial board composition across gender, geography, socioeconomic status, and geopolitical affiliations, raising critical ethical concerns. These disparities mirror patterns observed in other disciplines, including psychiatry, surgery, and anesthesia, where homogeneity in leadership undermines inclusivity and equitable representation in knowledge production [15–17]. This disparity across all four parameters in various disciplines demands prompt action. Therefore, it becomes important to address the homogeneity of editorial boards, as when a powerful center, which is based mainly on men belonging to the Global North and is in charge of building a global and/or international health narrative.

Gender imbalance persists as a defining feature: 66% of EB members are male, with journals such as the *Indian Journal of Leprosy* (92.3% male) and *Leprosy Review* (58.3% female) illustrating extremes. While some journals, such as *Current Tropical Medicine Reports*, achieve near parity, the underrepresentation of women in tropical medicine journals reflects systemic barriers. Consistent with our findings, previous evaluations of editorial board composition in hematology, psychiatry, surgery, neurology, nephrology, and urology journals have likewise revealed a pronounced underrepresentation of women, underscoring that gender imbalance in editorial leadership is a prevalent issue across medical specialties [15, 16, 23–25]. Women, often attributed to caregiving responsibilities and clinical roles, do not negate their potential editorial contributions [16, 23, 26]. Homophily in recruitment practices and implicit biases in selection processes perpetuate this gap, sidelining perspectives critical to addressing gendered health disparities, particularly in LMICs [27, 28].

This study highlights over half of the editorial members from HICs, while LICs constitute a mere 2.9%. In comparing our results to diversity analyses in other healthcare fields, Yip and Rashid [20] documented restricted regional representation on medical education journal boards, and Bould et al. (2022) reported similar limitations in the editorial boards of anesthesia journals [17, 29]. This skewing misaligns with global disease burdens: 85% of the world’s population resides in LMICs/LICs, yet their voices are marginalized in setting research agendas [30–32]. This imbalance risks creating a “Western outlook,” where priorities of HICs overshadow diseases of poverty, as seen in historical biases against neglected tropical diseases [33, 34].

Political-economic affiliations reveal a concentrated influence: 40.3% of editorial board members belong to the G7 countries, and 67.1% belong to the G20 countries. Such dominance undermines the principle of “respect for Southern innovation,” as knowledge from LMICs is frequently undervalued [35, 36]. Also this dominance has significant negative consequences as it hinders the recognition and dissemination of valuable research but also contributes to the development of solutions and policies that are often less effective. The COVID-19 pandemic starkly illustrated the consequences of excluding local expertise: lockdown protocols designed for high-resource settings faltered in LMIC contexts, exacerbating health inequities [37].

We propose three solutions to this inequity. First, structural policies must mandate diversity, equity, and inclusion (DEI) for gender, geographic, and income representation in high-impact journals to ensure equitable participation. Second, mentorship programs should be established to support researchers from LMICs through training and inclusive language policies, thereby reducing systemic barriers to entry. Third, transparent recruitment processes must be implemented to address implicit biases, including standardized selection criteria and the diversification of senior editorial roles, which play important roles in shaping future board composition.

### Study implications

Journals must adopt intersectional policies to advance diversity, equity, and inclusion (DEI) in addressing historical inequities. Prioritizing single dimensions of representation risks replacing one injustice with another. Holistic strategies centering marginalized voices are imperative to ensure that justice aligns with lived realities and systemic change. It is also important to ensure contextually relevant solutions and ethical knowledge dissemination. It demands dismantling the power structures that fuel inequality and establishing a research environment that values everyone’s expertise. Additionally, true decolonization requires a fundamental shift in mindset and a commitment to authentic collaboration and co-creation of knowledge.

One limitation of this study is its narrow scope in assessing gender diversity, as the analysis does not include nonbinary individuals due to data and methodological constraints. While we advocate that future research incorporate broader gender disaggregation, this is not feasible here. Additionally, our reliance on the NLM database and publicly available data may have resulted in the omission of relevant journals, particularly those addressing tropical diseases as a subdomain. For some editorial members, data were incomplete or unavailable, necessitating cross-referencing multiple sources to ensure accuracy. Furthermore, the study did not examine the representation of Indigenous and Adivasi communities, whose inclusion in the editorial board is vital for equitable governance. These gaps highlight the need for more comprehensive data collection and inclusive methodologies in future research. We acknowledge that our inclusion criteria focusing on journals with a global scope and explicit references to tropical or parasitic diseases in their titles may have excluded newer or regional publications. This may lead to a distorted view of editorial board composition by underrepresenting emerging local voices. Additionally, our study did not differentiate between journals with a primary clinical focus and those centered on laboratory-based research, which could further affect observed diversity patterns. These limitations highlight the need for future research to include region-specific journals and to distinguish between clinical and laboratory-based journals to capture a more localized, inclusive, and nuanced perspective on editorial governance in the Tropical medicine domain.

## Conclusion

The homogeneity of tropical medicine editorial boards threatens global bioethics, perpetuating epistemic injustice and stifling innovation. A diverse editorial board is not symbolic but essential for aligning research priorities with the needs of the populations most affected by tropical diseases. Without systemic reforms, the cycle of exclusion will persist, compromising scientific progress and ethical accountability Strategies such as instituting DEI (Diversity, Equity and Inclusion), creating targeted mentorship programs for LMIC researchers, and enforcing transparent, bias-resistant recruitment practices are important As gatekeepers of knowledge, journals must prioritize inclusivity to dismantle colonial legacies and ensure that marginalized voices actively lead and shape the discourse in tropical medicine and global health.

## Supplementary Information

Additional file 1.

## Data Availability

No datasets were generated or analysed during the current study.
