# Supplementary material for: Power imbalances in tropical medicine journals: an analysis of editorial board representation
Source: Trop Med Health. 2025 Jul 11;53:92. doi: 10.1186/s41182-025-00752-2 (PMC12247218; doi:10.1186/s41182-025-00752-2)
Supplement: Supplementary file 1 — Additional file 1. [file 41182_2025_752_MOESM1_ESM.docx]

**Supplementary Table 1: Search strategy to retrieve the relevant journals**

| **Search strategy** | **Hits** |
| --- | --- |
| “("Neglected tropical disease"[MeSH Terms] OR (Internationality [MeSH Terms] OR “Tropical medicine” [All Fields] OR NTD [All Fields]) AND ncbijournals)” | 153 |

**Supplementary Table 2: List of journals excluded in the second phase**

| **Name of Journal** | **Reason of exclusion** |
| --- | --- |
| Acta Amazonica | Regional scope |
| Anais do Instituto de Higiene e Medicina Tropical | Not in English and regional scope |
| Annales Academiae Medicae Stetinensis | Not a Tropical medicine journal |
| Archives of clinical infectious diseases | Not a Tropical medicine journal |
| The Central African journal of medicine | Regional scope |
| Journal of Microbes & Their Vectors Causing Human Infections | Not a Tropical medicine journal |
| Revista da Sociedade Brasileira de Medicina Tropical | Regional scope |
| The Southeast Asian journal of tropical medicine and public health | Regional scope |
| Malaria journal | Not a Tropical medicine journal |
